# Supplementary material for: Pair bond endurance promotes cooperative food defense and inhibits conflict in coral reef butterflyfish
Source: Sci Rep. 2018 Apr 19;8:6295. doi: 10.1038/s41598-018-24412-0 (PMC5908845; doi:10.1038/s41598-018-24412-0)
Supplement: Supplementary file 3 — Supplementary information [file 41598_2018_24412_MOESM3_ESM.pdf]

Supplementary Information

Pair bond endurance promotes cooperative food defense and inhibits conflict in coral reef butterflyfish

Authors:

Jessica P. Nowicki[1,2]*, Stefan P. Walker[1], Darren J. Coker[1,3], Andrew S. Hoey[1], Katia J. Nicolet[1], Morgan S. Pratchett[1]*

Affiliations:

[1]ARC Centre of Excellence for Coral Reef Studies, James Cook University, Townsville, QLD 4810, Australia
[2]Biology Department, Stanford University, Stanford, CA 94305, USA
[3]Red Sea Research Center, Division of Biological and Environmental Science and Engineering, King Abdullah University of Science and Technology, Thuwal, 23955-6900, Saudi Arabia

***Corresponding authors:**

Jessica Nowicki
E-mail: jnowicki@stanford.edu

Morgan Pratchett
E-mail : morgan.pratchett@jcu.edu.au

Supplementary Table S1. Taxa hypothesized to pair bond for assisted resource defense (ARD) purposes.

Taxon	(Resource type)<br>Evidence for assisted resource defence (ARD)*	Mode of ARD	Reason(s) for ARD	Partner fidelity	Reason(s) for partner fidelity
**Mammals**					
Eulemur rubriventer	(Food)[21]<br>1. Pairs stable and persist without reproductive activity[21]<br>2. Pair bond frequency and/or pair territoriality varies with resource availability[21]<br>3. Pairs work together or separately to defend resources[21]	Mutual[21]	Unknown	≥ 6 years[21]	Unknown
Lavia frons	(Food)[63]<br>1. Pairs stable and persist without reproductive activity[63]<br>2. Pairs work together or separately to defend resources[63]	Male-exclusive[63]	Improve energy budget of pair (untested)[63]	≥ 1 year[63]	Unknown
Castor fiber	(Food)[64]<br>1. Pairs stable and persist without reproductive activity[64]<br>2. Pairs work together or separately to defend resources[64]	Male-prioritized[64]	Secure food for females and offspring (untested)[64]	Long-term[64]	Unknown
**Birds**					
Anser anser	(Food)[65]<br>1. Pairs stable and persist without reproductive activity[65]<br>2. Pairs work together or separately to defend resources[65]	Mutual[65]	Improve competition, feeding, and survival (tested, supported)[65]	Long-term[62]	Unknown
Peucaea ruficauda	(Food, water)[81]<br>1. Pairs stable and persist without reproductive activity[81]	Female-prioritized[81]	Unknown	≥ 1 year[81]	Unknown
Branta leucopsis	(Food, nesting sites)[56]<br>1. Pairs stable and persist without reproductive activity[56]<br>2. Pairs work together or separately to defend resources[56]	Male-prioritized[56]	Improve feeding, energy reserve, and reproduction in females (feeding tested, supported)[56]	1 year- life-long[56]	Improves cooperative food acquisition and reproduction of pair (tested, supported)[56]
**Fish**					
Eretmodus cyanostictus	(Food, shelter)[66]<br>1. Pairs stable and persist without reproductive activity[66]<br>2. Pairs work together or separately to defend resources[66]	Male-prioritized[67]	Increase territory acquisition in females (tested, supported)[66]	Long-term[67]	Unknown

Chaetodon chrysurus (= *paucifasciatus*)	(Food)[11]<br>1. Pairs stable and persist without reproductive activity[11]<br>2. Pairs work together or separately to defend resources[11]	Mutual[11]	Reduce territory defence and improve feeding (tested, supported)[11]	Months-years[11]	Unknown
Chaetodon multicinctus	(Food)[40,61]<br>1. Pairs stable and persist without reproductive activity[40,61]<br>2. Pairs work together or separately to defend resources[40,61]	Male-prioritized [40,61]	Reduce territory defence in both sexes and improve feeding in female (tested, supported)[40,61]	Months-years[36]	Unknown
Chaetodon quadrimaculatus	(Food)[40.61]<br>1. Pairs stable and persist without reproductive activity[40,61]<br>2. Pairs work together or separately to defend resources[40,61]	Male-prioritized [40,61]	Reduce territory defence in both sexes and improve feeding in female (tested, supported)[40,61]	1 year– long-term[40]	Unknown
Chaetodon lunulatus	(Food)[**]<br>1. Pairs stable and persist without reproductive activity [**]<br>2. Pairs work together or separately to defend resources [**]	Mutual [**]	Improve feeding and energy reserves in both partners (tested, supported) [**]	≥ 7 years[68]	Improves cooperative food defence and reduces conflict between partners (tested, supported) [**]
Chaetodon baronessa	(Food)[**]<br>1. Pairs stable and persist without reproductive activity [**]<br>2. Pairs work together or separately to defend resources [**]	Male-prioritized [**]	Improve feeding and energy reserves in both partners (tested, supported) [**]	≥1.5 months[44]	Improves cooperative food defence and reduces conflict between partners (tested, supported) [**]
**Invertebrates**					
Hemilepistus reaumuri	(Burrow)[19,69]<br>1. Pairs stable and persist without reproductive activity[19,69]<br>2. Pairs work together or separately to defend resources[19,69]	Male-prioritized [19]	Females forage without losing burrow (untested)[19]	Unknown	Unknown

Alpheus angulatus	(Burrow)[23]<br>1. Pairs work together or separately to defend resources[23]	Male-prioritized [23]	Reduces risk of female eviction (tested, supported)[23]	Unknown	Unknown

Notes: *Predictions of ARDH for pairing put forth by [3,21] , ** Findings from current study

Supplementary Table S2. Partner fidelity among pairing species of butterflyfish.

Family and Genus	Species	Duration of partner fidelity*	Location	Ref.
Chaetodontidae				
Chaetodon				
	C. chrysurus	3 years	Sinai cst, Red Sea, Egypt	11
	C. fasciatus	6 years	Sinai cst, Red Sea, Egypt	11
	C. baronessa	1.5 months	Lizard Island, GBR, Australia	44
	C. lunulatus	1.5 months	Lizard Island, GBR, Australia	44
	C. lunulatus	6 months	Kuroshima Isl., Japan	31
	C. lunulatus	4 months	Heron Isl., GBR, Australia	42
	C. lunulatus	7 years	Heron Isl., GBR, Australia	68
	C. multicinctus	> 7 months	Kona cst, Hawaiian Isls., USA	36
	C. multicinctus	> 4 years	Hawaiian Isl., USA	33
	C. unimaculatus	1 year	Eniwetok At., GBR, Australia	42
	C. ornatissimus	1 year	Kona cst, Hawaiian Isls., USA	36
	C. quadrimaculatus	1 year	Kona cst, Hawaiian Isls., USA	36
	C. vagabundus	1.5 months	Lizard Island, GBR, Australia	49
Heniochus				
	H. intermedius	3 years	Sinai coast, Red Sea, Egypt	11

*In each case, the duration of partner fidelity equals the duration of the study, and therefore should be considered a minimum value.

Supplementary Table S3. Enduring vs. new partnerships: Standardized canonical coefficients (SCC) between canonical discriminant function (CDF_1) and response variables of *C. lunulatus* and *C. baronessa* to relationship phase (enduring vs. new partner) and day (day 1-5 = enduring partner; day ≥ 6 = new partner).

Response variable	Phase	Day
	CDF_1	CDF_1
	SCC	SCC
C. lunulatus		
Cohesive swimming rate	-0.37	-0.43
Within-pair aggression rate	0.61	0.61
Aggression per competitor rate	0.21	0.12
Feeding bite rate	-0.28	-0.29
Variance explained (%)	100	100
C. baronessa		
Cohesive swimming rate	0.56	0.60
Within-pair aggression rate	0.90	0.84
Aggression per competitor rate	0.19	0.11
Feeding bite rate	0.22	0.1
Variance explained (%)	100	100

Supplementary Table S4. Enduring vs. new partnerships: Means of standardized canonical scores of the first canonical discriminant function (CDF_1) for *C. lunulatus* and *C. baronessa* in response to relationship phase (with enduring partner vs. with new partner) and days (day 1-5 = with enduring partner; day ≥ 6 = with new partner).

	C. lunulatus **Mean**	***C. baronessa*** **Mean**
Phase		
Enduring	-0.63	-0.67
New	0.45	0.41
Day		
1	-0.66	-0.57
2	-0.62	-0.51
3	-0.67	-0.56
4	-0.53	-0.75
5	-0.61	-0.88
6	2.01	2.51
7	1.09	1.23
8	0.56	0.22
9	0.46	0.72
10	-0.01	-0.37
11	-0.55	-0.12
12	-0.48	-0.44
13	--	-0.23
14	--	-0.61

Supplementary Table S5. Enduring vs. new partnerships: Tests of between-subjects effects, with day in partnership for each relationship phase as a fixed factor.

	**df**	**MS**	**F**	**P**
C. lunulatus				
Enduring partnership				
Coordinated swimming rate	4	422.31	0.56	0.70
Within-pair aggression rate	4	0.09	1.46	0.23
Aggression per competitor rate	4	1.02	1.38	0.26
Feeding bite rate	4	229.19	0.24	0.92
New partnership				
Coordinated swimming rate	6	3029.10	3.67	0.00*
Within-pair aggression rate	6	30.47	3.06	0.01*
Aggression per competitor rate	6	7.80	1.14	0.35
Feeding bite rate	6	2498.50	2.51	0.03*
C. baronessa				
Enduring partnership				
Coordinated swimming rate	4	307.42	.73	0.58
Within-pair aggression rate	4	.020	1.000	0.42
Aggression per competitor rate	4	9.83	.86	0.50
Feeding bite rate	4	373.18	.36	0.83
New partnership				
Coordinated swimming rate	8	1665.500	2.030	.054

Within-pair aggression rate	8	1665.500	2.030	.054
Aggression per competitor rate	8	12.228	.554	.812
Feeding bite rate	8	2502.851	2.550	.017*

*significant differences within a testing group at alpha = 0.05.

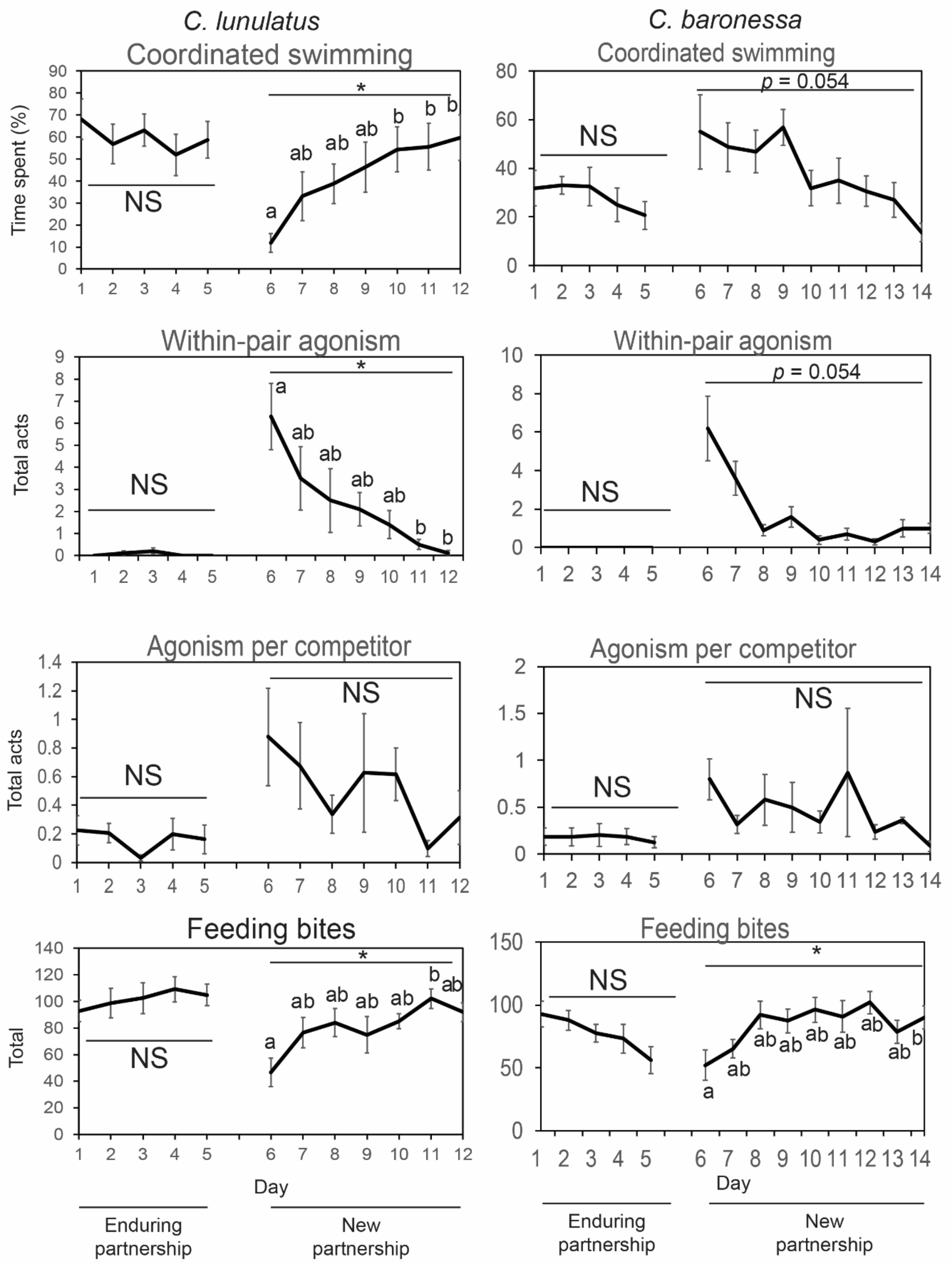

Supplementary Figure S1. Enduring vs. new partnerships: Profile plots of changes in behaviours in response to re-pairing and subsequent endurance of new partnerships throughout several days. Data are represented as the mean ± standard error; asterisks (*) represent a significant difference by MANOVA across days for each relationship phase (Enduring partnership; New partnership). Days not labelled with the same letter are significantly different as per Tukey's HSD *post hoc* test.

Individual 1

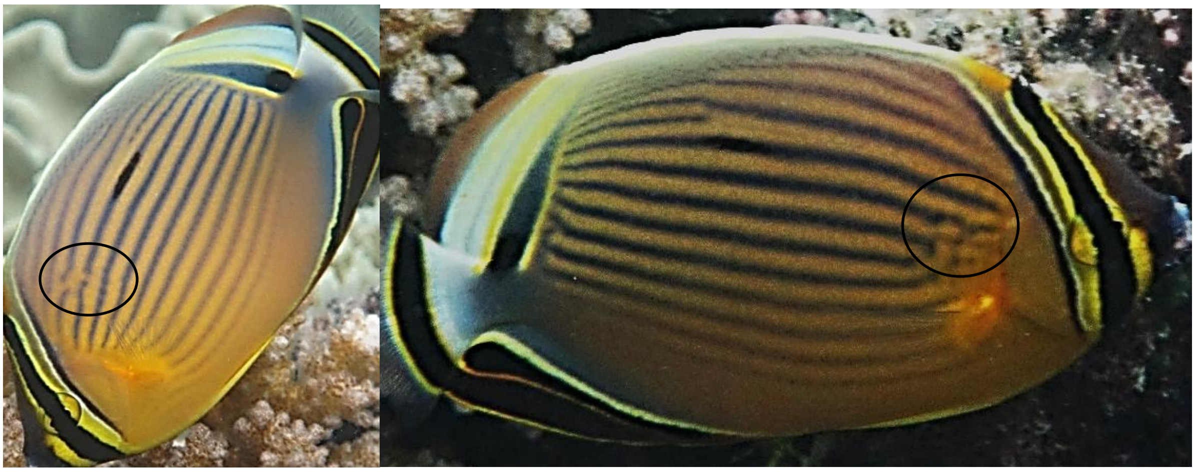

Individual 2

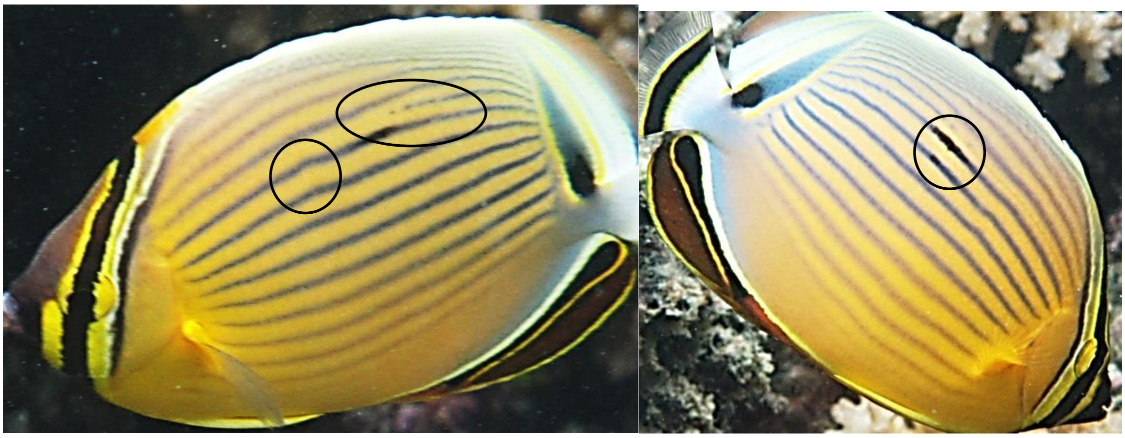

Individual 3

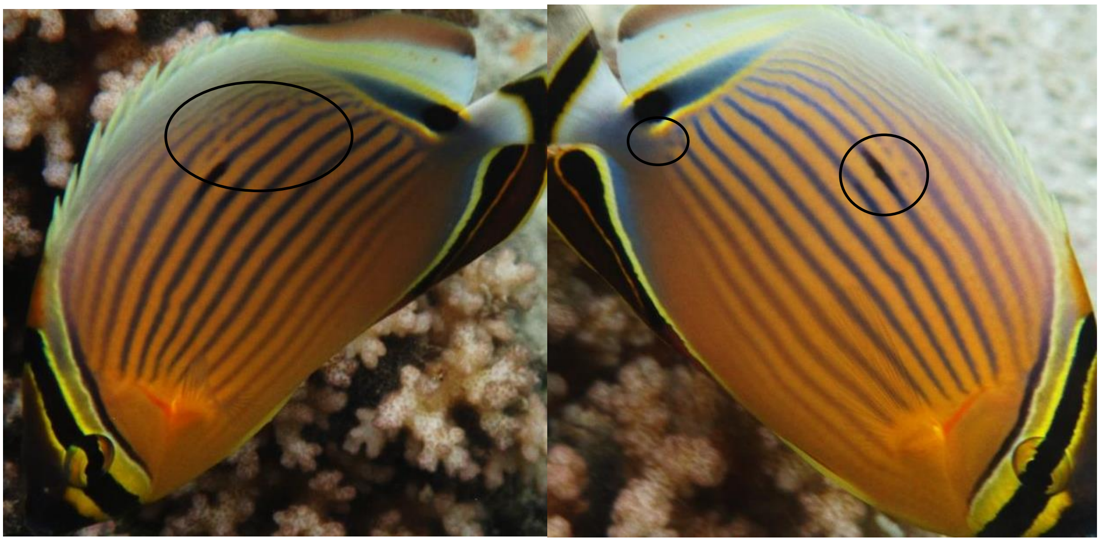

Individual 4

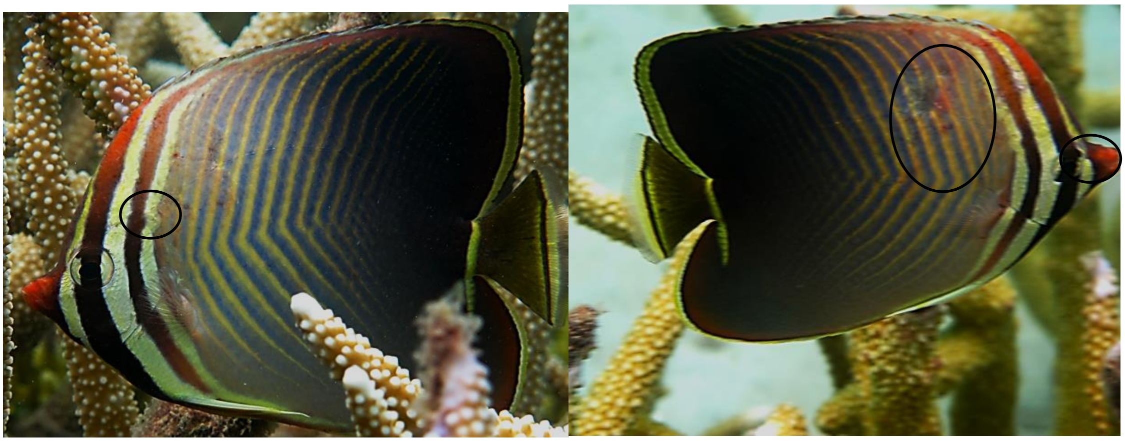

Individual 5

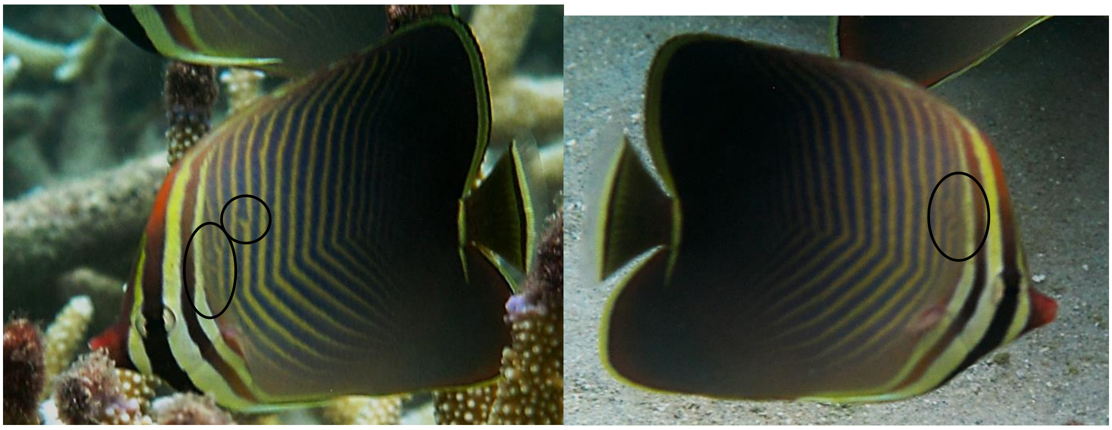

Individual 6

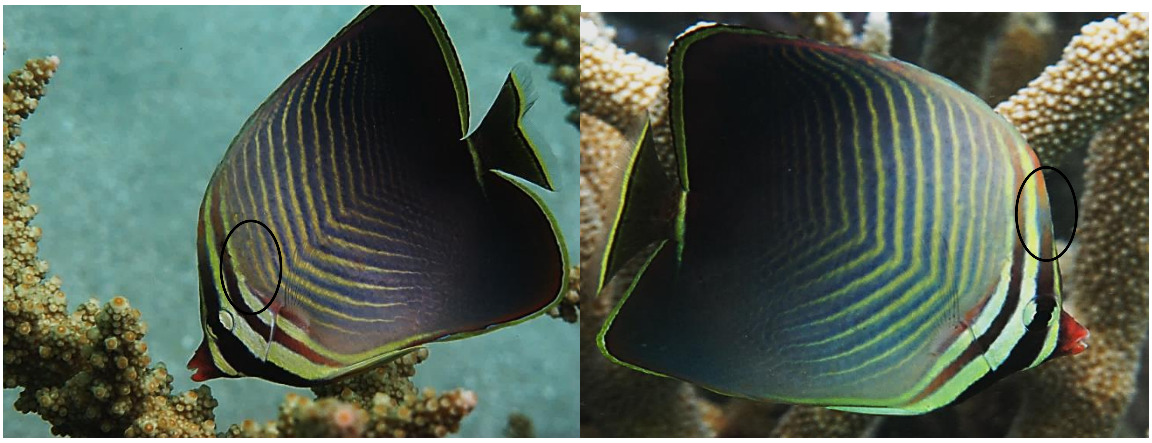

Supplementary Figure S2: Examples of photographs taken of *C. lunulatus* and *C. baronessa*, highlighting sets of naturally-occurring features on right and left sides of the body that uniquely distinguish each individual. Pictures were printed on paper, laminated, and taken underwater with observers to identify and monitor focal fishes throughout partner removal study.

Video S1: A naturally occurring pair of *C. lunulatus* displaying characteristic cohesive pair swimming throughout their feeding territory.

Video S2: An experimentally-induced new pair (≤ 18hr persistence) of *C. lunulatus* displaying reduced pair swimming and heightened intra- and inter-pair aggression, resulting in reduced feeding bites.
